# Early and Late Readmissions of Radiation Proctitis in the United States: Are We Getting Better?

**DOI:** 10.3390/jcm13020423

**Published:** 2024-01-12

**Authors:** Dushyant Singh Dahiya, Bhanu Siva Mohan Pinnam, Michelle Ishaya, Saurabh Chandan, Manesh Kumar Gangwani, Sahib Singh, Hassam Ali, Amir Humza Sohail, Andrew Canakis, Daryl Ramai, Christina Zelt, Sumant Inamdar, Mohammad Al-Haddad, Mariajose Rojas-DeLeon, Neil R. Sharma

**Affiliations:** 1 Division of Gastroenterology, Hepatology & Motility, The University of Kansas School of Medicine, 2000 Olathe Blvd, Kansas City, KS 66160, USA; 2 Department of Internal Medicine, John H. Stroger Jr. Hospital of Cook County, Chicago, IL 60612, USA; 3 Division of Gastroenterology and Hepatology, CHI Creighton University Medical Center, Omaha, NE 68178, USA; 4 Department of Internal Medicine, The University of Toledo, Toledo, OH 43606, USA; 5 Department of Internal Medicine, Sinai Hospital, Baltimore, MD 21215, USA; 6 Division of Gastroenterology, Hepatology and Nutrition, East Carolina University/Brody School of Medicine, Greenville, NC 27858, USA; 7 Complex Surgical Oncology, Department of Surgery, University of New Mexico, Albuquerque, NM 87106, USA; 8 Division of Gastroenterology and Hepatology, University of Maryland School of Medicine, Baltimore, MD 21201, USA; 9 Division of Gastroenterology and Hepatology, The University of Utah School of Medicine, Salt Lake City, UT 84112, USA; 10 Interventional Oncology & Surgical Endoscopy (IOSE) Division, GI Oncology Tumor Site Team, Parkview Cancer Institute, Parkview Health, Fort Wayne, IN 46845, USA; 11 Division of Gastroenterology and Hepatology, University of Arkansas for Medical Sciences, Little Rock, AR 72205, USA; 12 Division of Gastroenterology and Hepatology, Indiana University School of Medicine, Indianapolis, IN 46202, USA; 13 Division of Gastroenterology and Hepatology, Parkview Health System, Fort Wayne, IN 46845, USA

**Keywords:** radiation proctitis, outcomes, readmission, cost, mortality

## Abstract

**Background/Aims:** Radiation proctitis (RP), a well-known complication of pelvic radiation therapy, may lead to recurrent hospitalizations. We aimed to assess readmissions of RP in the United States. **Methods:** We analyzed the Nationwide Readmission Database from 2016 to 2020 to identify all 30-, 60-, and 90-day readmissions of RP in the United States. Hospitalization characteristics, predictors, clinical outcomes, and healthcare burdens were assessed. **Results:** From 2016 to 2020, we noted a declining trend of 30-, 60-, and 90-day readmissions of RP in the US. However, the all-cause 30-, 60-, and 90-day readmission rates of RP were still high at 13.7%, 19.4%, and 23.16%, respectively. On readmission, RP was identified as the admitting diagnosis in only 20.61%, 17.87%, and 15.76% of 30-, 60-, and 90-day readmissions, respectively. The mean age for all readmissions was 70 years with a significant male dominance. Lower endoscopy at index admission reduced the risk of readmissions within 90 days, but this was not statistically significant. However, the Charlson Comorbidity Index (CCI) score was an independent predictor of all readmissions. Furthermore, the mean length of stay was 5.57 (95% CI 5.15–6), 5.50 (95% CI 5.12–5.89), and 5.47 (95% CI 5.07–5.87) days and the mean hospitalization charge was USD 60,451 (95% CI USD 54,728–66,174), USD 62,671 (95% CI USD 57,326–68,015), and USD 62,144 (95% CI USD 57,144–67,144) for 30-, 60-, and 90-day readmissions. The all-cause inpatient mortality for 30-, 60-, and 90-day readmissions was 3.58%, 3.89%, and 3.46%, respectively. **Conclusions:** RP readmissions are a significant healthcare burden. Further efforts must be directed toward improving management strategies to reduce readmission rates.

## 1. Introduction

Radiotherapy (RT) is an essential therapeutic tool in managing numerous pelvic malignancies involving the genitourinary, gastrointestinal, and gynecologic systems. Improved radiation delivery techniques, earlier detection of malignancies, and the introduction of novel chemotherapeutic agents have translated into a promising increase in survival rates for pelvic malignancies [1,2]. Despite these advances, radiation-related adverse events continue to pose a major challenge in this subset population. Gastrointestinal (GI) toxicity adds a significant burden to the morbidity and complications already associated with RT. One such GI toxicity that clinicians often encounter is radiation proctitis (RP).

RP occurs due to ionizing radiation-induced damage to the rapidly dividing epithelial cells in the gut. Exposure to RT leads to the creation of oxygen-free radicals which directly damage cellular proteins, DNA, and lipids, resulting in cellular necrosis. Although this damage represents a continuous spectrum of pathological changes in the gut mucosa in a time-dependent manner, there is some degree of overlap between the acute and chronic phases. The exact incidence and prevalence of RP is currently unknown. However, it is estimated that about 90% of patients will experience chronic RP within 2 years of RT [3,4].

RP significantly impacts patients’ overall quality of life and adds to the current healthcare burden. With the varying efficacy of the current treatment modalities, patients often experience recurrent hospitalizations. The current literature regarding readmissions in patients with radiation proctitis is fairly limited with only a few single-center studies evaluating the disease entity. National-level data regarding the readmission burden in this patient population are scarce. To improve survival rates in patients receiving RT for pelvic malignancies, understanding this crucial metric is essential. Hence, in this study, we aimed to evaluate hospitalization characteristics, readmission rates, clinical outcomes, and the healthcare burden of RP in the United States (US) at a national level.

## 2. Methods

### 2.1. Data Source

The data analyzed for this retrospective study were derived from the Nationwide Readmissions Database (NRD). NRD is the largest, publicly available, multi-ethnic readmission database in the US, maintained by the Agency for Healthcare Research and Quality (AHRQ) Healthcare Cost and Utilization Project (HCUP) State Inpatient Databases [5]. For each calendar year, NRD contains discharge information from geographically dispersed and diverse states. It contains a weighted sample of hospitalizations in the United States, which can be used to derive national estimates of hospitalizations. Within the database, patients are tracked using unique identifier numbers that are not linked to patient or hospital data; hence, all data are de-identified to maintain patient privacy.

### 2.2. Study Population

In this study, we utilized the NRD from 2016 to 2020 to identify all hospitalizations with a principal diagnosis of RP using International Classification of Diseases 10 (ICD-10) codes. Individuals < 18 years of age, traumatic, and elective hospitalizations were excluded from the study. Hospitalizations in December, hospitalizations in November and December, and hospitalizations in October, November, and December were excluded from the 30-, 60-, and 90-day readmission analyses, respectively, as NRD does not use the same unique identifier for the subsequent year.

### 2.3. Statistical Analysis and Outcome Measures

The data were analyzed using Stata^®^ Version 18 software (StataCorp, College Station, TX, USA). All analyses were performed using weighted samples for national estimates in adjunct with HCUP regulations for utilizing the NRD. The comorbidity burden was quantified using the Charlson Comorbidity Index (CCI) scoring system. The CCI score is derived from 19 medical conditions and adjusts for variable morbidity rates within a single-cohort population. Each comorbidity category has a weight from 1 to 6, primarily based on the adjusted risk of mortality or resource use, and the cumulative sum of all the weights results in a single comorbidity score for a patient. In the scoring system, a CCI score of 0 indicates the absence of comorbidities. However, as the score increases, it becomes more likely that the associated outcome will result in higher mortality or increased resource utilization.

A univariate regression analysis was performed with the outcomes as the dependent variable and potential confounders as the independent predictor. A *p*-value of 0.2 was considered to imply a possible association, and these variables were adjusted for in the eventual multivariate regression model. A multivariate regression analysis was used to calculate the odds of all-cause readmission, inpatient mortality, length of stay (LOS), and total hospital charge (THC) after adjusting for age, gender, CCI category, type of insurance, mean household income, and hospital characteristics. THC from 2016 to 2020 was adjusted for inflation in the healthcare sector using the CPI inflation calculator maintained by the US Bureau of Labor Statistics. Multivariate linear and logistic regression were used to compare continuous and categorical variables, respectively. A 2-sided *p* < 0.05 was considered to represent statistical significance.

### 2.4. Ethical Considerations

The NRD lacks specific patient and hospital identifiers. Due to the deidentified nature of the study sample, this study was exempt from Institutional Review Board (IRB) approval as per guidelines put forth by our institutional IRB for NRDstudies.

## 3. Results

### 3.1. 30-Day Readmissions of Radiation Proctitis

The all-cause 30-day readmission rate was noted to be 13.7% with a mean age of 70.96 years. A majority of these patients were males (69.92%), belonged to the 65–79-year age group (49%), and had a CCI score ≥ 3 (62.89%). Hospitalization characteristics for 30-day readmissions are detailed in Table 1.

**Figure 1:**
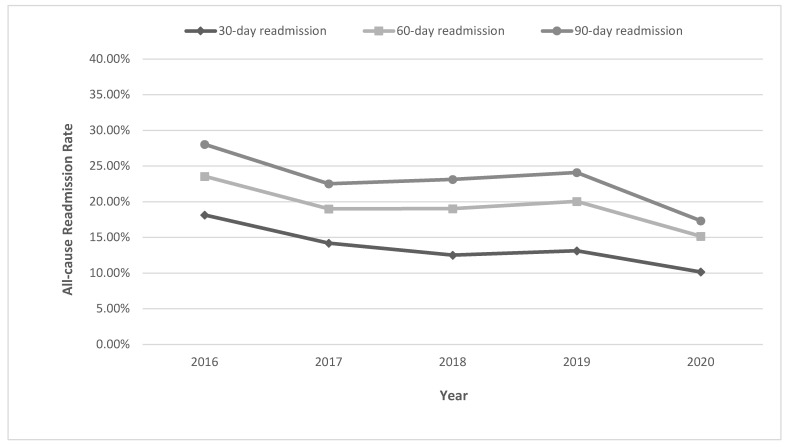
Yearly trends for 30-day, 60-day, and 90-day readmissions of radiation proctitis in the United States.

From 2016 to 2020, we noted a declining trend for all-cause 30-day readmission of RP from 18.14% in 2016 to 10.16% in 2020 (*p*-trend < 0.001) (Figure 1). However, on readmission, RP was identified as the admission diagnosis in only 20.61% of patients (Table 2). Other common readmission diagnoses were GI bleeding (10.36%) and ulcer of the anus and rectum (3.54%).

CCI was identified to be a significant predictor of readmission with higher CCI increasing the odds of readmissions [CCI = 1: odds ratio (OR) 1.46, 95% confidence interval (CI) 1.0–2.11, *p* = 0.044; CCI = 2: OR 1.47, 95% CI 1.04–2.08, *p* = 0.027; CCI 3 or more: OR 1.9, 95% CI 1.4–2.57, *p* < 0.001; with CCI = 0 as the referent) (Table 3).

### 3.2. 60-Day Readmissions of Radiation Proctitis

For 60-day readmissions of RP, the readmission rate was noted to be 19.4%, and 47.15% of these patients belonged to the 65–79-year age group. Males constituted 69.83% of these readmissions and a majority (61.59%) of readmissions had a CCI of 3 or more. Hospitalization characteristics for these 60-day readmissions are detailed in Table 1.

Overall, we noted a decreasing trend for the all-cause 60-day readmission rate of RP from 23.56% in 2016 to 15.16% in 2020 (*p*-trend = 0.001) (Figure 1). The most common principal diagnosis on 60-day readmission was radiation proctitis (17.87%), followed by GI bleeding (9.92%) and sepsis (5.12%) (Table 2).

CCI was a statistically significant predictor of 60-day readmission, with a CCI of 2 or more increasing the odds of readmission [CCI = 2: OR 1.53, 95% CI 1.13–2.05, *p* = 0.005; CCI 3 or more: OR 1.89, 95% CI 1.46–2.45, *p* < 0.001; with CCI = 0 as the referent) (Table 3).

### 3.3. 90-Day Readmissions of Radiation Proctitis

The all-cause 90-day readmission rate was 23.16%, and 46.38% of these patients were of the 65–79-year age group. Males made up 69.58% of 90-day readmissions. A CCI score ≥3 was noted for 62.92% of 90-day readmissions. Table 1 demonstrates hospitalization characteristics for 90-day readmissions of RP.

For all-cause 90-day readmissions, we noted a declining trend from 28.04% in 2016 to 17.33% in 2020 (*p*-trend < 0.001) (Figure 1). RP was identified as the principal admission diagnosis in only 15.76% of patients at 90-day readmission, while the other common principal admission diagnoses were GI bleeding (9.9%) and sepsis (4.78%) (Table 2).

The CCI score was identified to be an independent predictor of 90-day readmissions, with a CCI of 2 or more associated with higher odds of 90-day readmissions [CCI = 2: OR 1.59, 95% CI 1.19–2.12, *p* = 0.002; CCI 3 or more: OR 2, 95% CI 1.55–2.58, *p* < 0.001; with CCI = 0 as the referent) (Table 3).

### 3.4. Multiple Readmissions of Radiation Proctitis

Between 2016 and 2020, 8.04% and 5.58% of index hospitalizations were readmitted twice and more than twice within 30 days, respectively. Furthermore, 7.4% and 3.4% of index hospitalizations experienced two readmissions and more than two readmissions within 60 days, respectively. Within 90 days, 9.4% and 6.15% of index hospitalizations had two readmissions and more than two readmissions, respectively.

### 3.5. Healthcare Burden of Radiation Proctitis Readmissions

The mean LOS for 30-, 60-, and 90-day readmissions were 5.57 (95% CI 5.15–6), 5.50 (95% CI 5.12–5.89), and 5.47 (5.07–5.87) days, respectively (Table 4). The mean THC concurred at 30-, 60-, and 90-day readmissions were USD 60,451 (95% CI USD 54,728–66,174), USD 62,671 (95% CI USD 57,326–68,015), and USD 62,144 (95% CI USD 57,144–67,144), respectively (Table 4).

### 3.6. Lower Endoscopy and Its Impact on Radiation Proctitis-Specific Readmission Rate

**Figure 2:**
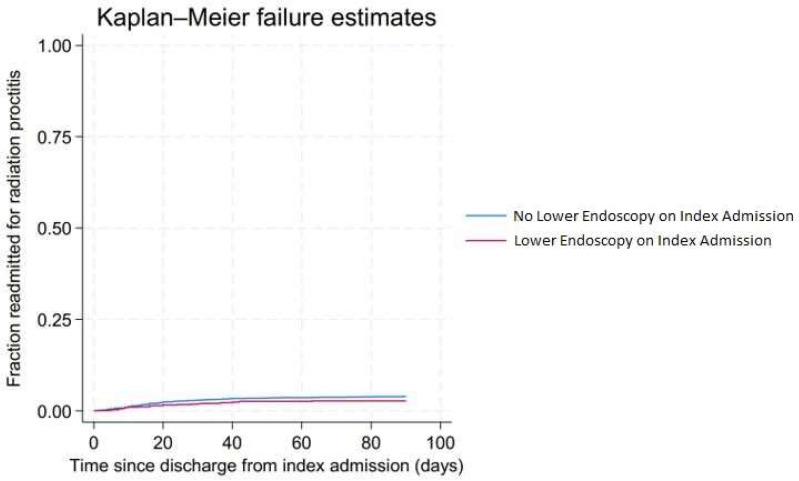
Kaplan–Meier curve for 90-day readmission for radiation proctitis stratified by colonoscopy at index admission.

On index admission, 18.82% of patients received lower endoscopy. Furthermore, 10.33%, 9.14%, and 9.11% of the 30-, 60-, and 90-day readmissions received lower endoscopy, respectively. Lower endoscopy on index admission reduced the risk of 90-day readmission of RP, but this was not statistically significant (hazard ratio (HR): 0.66, 95% CI 0.42–1.05, *p* = 0.083). Figure 2 demonstrates the Kaplan–Meier curve for 90-day readmission for radiation proctitis stratified by colonoscopy at index admission.

### 3.7. All-Cause Inpatient Mortality of Radiation Proctitis Readmissions

The all-cause inpatient mortality rate for the 30-, 60-, and 90-day readmissions were 3.58%, 3.89%, and 3.46%, respectively. Further stratification of the mortality rate is detailed in Table 5.

## 4. Discussion

Our study highlights the high readmission rates observed in patients with RP in the US despite it being a well-known clinical entity. Our findings indicate that up to 23% of index admissions experience readmission for RP within the 90-day period. Often, not all patients are identified to have a readmission diagnosis of RP. In fact, on readmission, RP was identified as the admitting diagnosis in only 20.61%, 17.87%, and 15.76% of 30-, 60-, and 90-day readmissions, respectively. Although we noted a higher proportion of males compared to females on all readmissions, gender did not have a statistically significant impact on readmission rates. Increasing CCI score was an independent predictor of all readmissions. Furthermore, lower endoscopy on index admission reduced the risk of 90-day readmission of RP, but this was not found to be statistically significant. From a healthcare burden perspective, RP is associated with significant hospital charges and length of hospital stay. The overall trend of readmission rates of RP was on a decline from 2016 to 2020 and is expected to decline further, thereby reducing the healthcare burden. However, this must be interpreted with caution as the COVID-19 pandemic may have, in part, had a role to play in lower 30-, 60-, and 90-day readmissions of RP in 2020, thereby contributing to the declining trend.

Over the last few decades, RT has undergone a revolutionary change. Numerous studies have reported increasing utilization of targeted RT for genitourinary cancers, while the use of conventional RT has either plateaued or declined [6,7,8,9]. The most notable shift has been towards the use of targeted intensity-modulated RT (IMRT). This method of radiation delivery allows for higher doses of radiation to be delivered to target tissues while simultaneously reducing the incidence of severe gastrointestinal toxicity compared to conventional RT [6]. These changes reflect the results of continued innovation and development in the modes of radiation delivery to reduce adverse events and hospitalization rates. In the current literature, there is limited data on readmission rates of RP worldwide and in the US. In a study of patients with prostate cancer receiving RT, RP, and colitis accounted for 46.3% of all readmissions during a median follow-up period of 5.1 years [10]. However, in our study, despite having a larger sample size, the all-cause 30-, 60-, and 90-day readmission rate of RP was 13.7%, 19.4%, and 23.16%, respectively. Although these rates are lower than that reported in the current literature, possibly due to a shift towards targeted RT, it is still alarming. Additionally, only about one-fifth of all readmissions had an admitting diagnosis of RP. Limited available therapeutic options, challenging diagnosis and management on index hospitalization, the persistence of bothersome symptoms, and resistant disease course may, in part, explain the high readmission rates.

From a gender perspective, we noted a significant male predominance for all readmissions of RP in the US. This can, in part, be attributed to the distribution of RT-treated cancers. As per the most recent statistics available from the US Centers for Disease Control and Prevention (CDC), there is a much higher incidence of prostate cancer (116.6 per 100,000 men) relative to uterine (28.1 per 100,000 women) and cervical (7.7 per 100,000 women) cancers, which may require RT [11]. However, in our analysis, gender was not identified to be an independent predictor for readmissions of RP. Hence, additional large, prospective, multicenter studies are needed to fully understand and investigate the impact of gender on RP.

Similar to previous studies, we identified that a higher comorbidity burden (high CCI score) was a significant independent predictor of all readmissions for RP. Higher comorbidity is often associated with a greater severity of disease, which places these individuals at a higher risk of hospitalization and readmission [12]. Furthermore, studies have shown that RP patients may require multiple sessions of endoscopic therapy (ranging between 1 and 8) to adequately treat the clinical symptoms of RP [13,14,15,16,17]. However, no study has truly assessed the impact of lower endoscopy in reducing the readmission burden of RP. This may be due to the fact that to evaluate the utility of endoscopic therapy for RP patients in randomized controlled trials, the control group would require withholding of such therapy, which is a deviation from the standard of care and unethical. Ours is the first study in the US, on a national level, which investigated the impact of lower endoscopy on all readmissions of RP. We noted that lower endoscopy on index admission does not statistically reduce readmission rates of RP. This important real-world data can help gastroenterologists in the complex decision-making process while managing RP patients. Furthermore, it can help patients make a more informed decision regarding their care.

Cancers requiring pelvic RT have been on the rise in the US. The incidence of prostate cancer, once noted to have declined in the early 2000s after instituting screening tests in preventive health measures, has experienced a steep climb over the last decade [18]. Endometrial cancer and rectal cancer have also been on the rise, with studies suggesting a shift towards a younger age at diagnosis [19,20,21]. These changes in incidence reflect increased utilization of RT, thereby further increasing healthcare resource utilization and overall burden. In our study, the mean LOS for readmissions of RP was about 5.5 days and the mean THC associated with each admission was around USD 60,000. The mortality rate for 30-, 60-, and 90-day readmissions of RP were 3.58%, 3.89%, and 3.46%, respectively. Early diagnosis on index admission and readmission, appropriate risk stratification, medical optimization prior to discharge, and meticulous outpatient follow-up could help reduce the overall morbidity, mortality, and healthcare burden associated with RP in the US.

Our study has numerous strengths and some limitations. A key strength of our study is the study population, which has been derived from one of the largest and most racially diverse inpatient databases in the US—the NRD. Hence, the results of our study are applicable to a large sample of index hospitalizations and readmissions in the US. The NRD stores information on up to 25 procedures and 40 diagnosis codes per admission, minimizing the risk of under-representation of comorbidities. However, we do acknowledge the limitations associated with our study. The NRD database lacks information on the total number of episodes of RP in each individual patient, time from RT to development of RP, hospital course, treatment aspects, pharmacological management, and post-discharge course of these patients. As the NRD stores data in the form of ICD codes rather than patient information to protect patient confidentiality, we were unable to further assess the exact etiology for GI bleeding, ulcers of the anus and rectum, and ‘others’ listed in Table 2. The NRD also reports only all-cause inpatient mortality rates, rather than individual mortality rates for the clinical entity itself or its complications, and it does not contain procedural details for the clinical entity. Furthermore, due to the retrospective nature of the study design, all biases associated with retrospective studies are applicable. Lastly, the NRD is an administrative database maintained through data collection organizations that use the ICD coding system to store inpatient data. Hence, the possibility of human coding errors cannot be excluded. However, despite these limitations, we believe that the large sample size and a comprehensive analysis technique help us better understand the readmission patterns associated with RP in the US.

## 5. Conclusions

Pelvic organ malignancies are effectively treated with RT. With the advent of improved radiation delivery techniques, there has been a notable increase in survival rates. However, radiation proctitis remains a significant concern due to the high readmission rates at 30, 60, and 90 days after hospitalization, leading to a significant burden on the healthcare system in the US. The CCI score is an important predictor of all-cause readmission. However, endoscopy at index admission did not reduce readmission rates. To reduce readmission rates, medical comorbidities should be optimized in high-risk patients. A multidisciplinary approach to care that includes medical oncologists, radiation oncologists, therapeutic endoscopists, primary care physicians, and patient education is crucial. Given the increasing incidence of pelvic cancers, continuous efforts must be made to improve this vital healthcare quality metric.

## Figures and Tables

**Table 1:** Hospitalization characteristics for 30-day, 60-day, and 90-day readmissions of radiation proctitis in the United States.

	30-Day Readmissions	*p*-Value *	60-Day Readmissions	*p*-Value *	90-Day Readmissions	*p*-Value *
Total Number	1429 (13.7%)		1796 (19.4%)		1912 (23.16%)	
Mean Age (years) ± Standard Error	70.96 (70.06–71.86)		70.83 (69.95–71.7)		70.78 (69.81–71.76)	
Age Groups						
18–34	14 (0.97%)	0.342	20 (1.09%)	0.236	27 (1.41%)	0.1004
35–49	70 (4.9%)		100 (5.55%)		108 (5.66%)	
50–64	271 (19.02%)		339 (18.9%)		359 (18.76%)	
65–79	700 (49%)		847 (47.15%)		887 (46.38%)	
≥80	373 (26.11%)		491 (27.3%)		531 (27.79%)	
Gender						
Male	999 (69.92%)	0.4	1254 (69.83%)	0.19	1331 (69.58%)	0.134
Female	430 (30.08%)		542 (30.17%)		582 (30.42%)	
Charlson Comorbidity Index (CCI)						
CCI = 0	144 (10.06%)	<0.001	191 (10.64%)	0.0004	192 (10.05%)	<0.001
CCI = 1	142 (9.96%)		180 (9.99%)		171 (8.95%)	
CCI = 2	244 (17.09%)		320 (17.78%)		346 (18.08%)	
CCI ≥ 3	899 (62.89%)		1106 (61.59%)		1203 (62.92%)	
Hospital Region						
Metropolitan	1345 (94.11%)	0.518	1689 (93.76%)	0.634	1796 (93.91%)	0.156
Micropolitan	69 (4.86%)		93 (5.19%)		92 (4.82%)	
Non-urban	14 (1.03%)		14 (0.77%)		24 (1.27%)	
Hospital Bed Size						
Small	226 (15.83%)	0.454	291 (16.2%)	0.636	329 (17.18%)	0.748
Medium	367 (25.66%)		480 (26.72%)		531 (27.76%)	
Large	836 (58.51%)		1025 (57.07%)		1053 (55.06%)	
Hospital Location and Teaching Status						
Metropolitan non-teaching	245 (17.15%)	0.384	328 (18.28%)	0.682	349 (18.27%)	0.606
Metropolitan teaching	1100 (76.96%)		1361 (75.76%)		1447 (75.64%)	
Non-metropolitan	84 (5.89%)		107 (5.96%)		116 (6.09%)	
Insurance						
Medicare	1087 (77.76%)	0.22	1368 (77.67%)	0.016	1446 (77.23%)	0.001
Medicaid	130 (9.34%)		175 (9.94%)		199 (10.6%)	
Private insurance	168 (12.03%)		209 (11.86%)		217 (11.57%)	
Self-pay	12 (0.87%)		9 (0.52%)		11 (0.6%)	

* non-significant *p*-value for demographics implies good randomization of the population analyzed.

**Table 2:** Principal diagnosis on 30-day, 60-day, and 90-day readmission for radiation proctitis hospitalization in the United States.

	Principal Diagnosis at 30-Day Readmission	Percentage	Principal Diagnosis at 60-Day Readmission	Percentage	Principal Diagnosis at 90-Day Readmission	Percentage
1	Radiation proctitis	20.61%	Radiation proctitis	17.87%	Radiation proctitis	15.76%
2	Gastrointestinal bleeding	10.36%	Gastrointestinal bleeding	9.92%	GI bleed	9.90%
3	Ulcer of anus and rectum	3.54%	Sepsis	5.12%	Sepsis	4.78%
4	Heart failure with chronic kidney disease	3.23%	Ulcer of anus and rectum	3.17%	Rectal malignancy	3.13%
5	Dehydration	1.32%	Heart failure with chronic kidney disease	2.81%	HF with CKD	3.11%
6	Other	60.94%	Other	64.53%	Other	63.32%

**Table 3:** Predictors for all-cause 30-day, 60-day, and 90-day readmissions of radiation proctitis in the United States.

Variable	30-Day Readmission aOR (95% CI)	*p*-Value	60-Day Readmission aOR (95% CI)	*p*-Value	90-Day Readmission aOR (95% CI)	*p*-Value
Gender						
Male	Referent					
Female	1.04 (0.85–1.28)	0.672	1.07 (0.89–1.29)	0.418	1.09 (0.91–1.30)	0.322
Age Groups						
18–34	Referent					
35–49	0.83 (0.33–2.09)	0.707	0.76 (0.33–1.7)	0.507	0.55 (0.26–1.15)	0.116
50–64	0.93 (0.33–2.16)	0.707	0.69 (0.33–1.41)	0.313	0.17 (0.22–1.01)	0.055
65–79	0.87 (0.36–2.06)	0.756	0.63 (0.29–1.35)	0.24	0.43 (0.20–0.91)	0.029
≥80	0.73 (0.30–1.76)	0.486	0.56 (0.26–1.22)	0.15	0.40 (0.18–0.86)	0.019
Charlson Comorbidity Index						
CCI = 0	Referent					
CCI = 1	1.46 (1–2.11)	0.044	1.33 (0.96–1.83)	0.078	1.20 (0.87–1.65)	0.261
CCI = 2	1.47 (1.04–2.08)	0.027	1.53 (1.13–2.05)	0.005	1.59 (1.19–2.12)	0.002
CCI ≥ 3	1.9 (1.40–2.57)	<0.001	1.89 (1.46–2.45)	<0.001	2 (1.55–2.58)	<0.001
Hospital Region						
Metropolitan	Referent					
Micropolitan	1.59 (0.31–7.99)	0.573	1.85 (0.51–6.61)	0.95	1.45 (0.79–1.52)	0.565
Non-urban	-	-	-	-	-	-
Hospital Bed Size						
Small	Referent					
Medium	0.94 (0.71–1.24)	0.676	0.98 (0.76–1.27)	0.919	0.96 (0.74–1.23)	0.764
Large	0.95 (0.75–1.21)	0.703	0.93 (0.74–1.16)	0.551	0.85 (0.68–1.05)	0.143
Hospital Location and Teaching Status						
Metropolitan non-teaching	Referent					
Metropolitan teaching	0.87 (0.71–1.07)	0.213	0.88 (0.73–1.06)	0.2	0.89 (0.74–1.07)	0.23
Non-metropolitan	0.39 (0.083–1.90)	0.249	0.39 (0.11–1.34)	0.138	0.49 (0.16–1.49)	0.215
Insurance						
Medicare	Referent					
Medicaid	0.98 (0.69–1.39)	0.926	1.02 (0.73–1.42)	0.903	1.09 (0.79–1.52)	0.565
Private insurance	0.99 (0.73–1.34)	0.974	0.93 (0.70–1.24)	0.658	0.88 (0.67–1.17)	0.401
Self-pay	1.26 (0.56–2.82)	0.565	0.84 (0.35–2.02)	0.704	0.83 (0.35–1.97)	0.677

aOR: adjusted odds ratio. CI: confidence interval. CCI: Charlson Comorbidity Index.

**Table 4:** Outcomes for 30-day, 60-day, and 90-day readmissions of radiation proctitis in the United States.

Outcome	30-Day Readmissions	60-Day Readmissions	90-Day Readmissions
Mean Total Hospitalization Charges (USD)	60,451 (54,728–66,174)	62,671 (57,326–68,015)	62,144 (57,144–67,144)
Mean Length of Stay (days)	5.57 (5.15–6.0)	5.50 (5.12–5.89)	5.47 (5.07–5.87)
Lower Endoscopy	147 (10.33%)	164 (9.14%)	174 (9.11%)
Readmitted Twice	8.04%	7.40%	9.40%
Readmitted More Than Twice	5.58%	3.40%	6.15%

**Table 5:** All-cause inpatient mortality 30-day, 60-day, and 90-day readmissions of radiation proctitis in the United States.

	30-Day Readmission	60-Day Readmission	90-Day Readmission
All-Cause Mortality			
Overall mortality	51 (3.58%)	70 (3.89%)	66 (3.46%)
Age groups			
18–34	0	0	0
35–49	1 (1.9%)	1 (1.3%)	1 (1.26%)
50–64	9 (3.2%)	7 (2.11%)	5 (1.33%)
65–79	24 (3.4%)	31 (3.77%)	30 (3.40%)
≥80	17 (4.46%)	29 (6%)	30 (5.63%)
Gender			
Male	41 (4.1%)	57 (4.58%)	51 (3.81%)
Female	10 (2.26%)	13 (2.28%)	16 (2.68%)
Charlson Comorbidity Index (CCI)			
CCI = 0	3 (2.07%)	3 (1.5%)	3 (1.55%)
CCI = 1	2 (1.14%)	2 (0.9%)	2 (0.94%)
CCI = 2	4 (1.5%)	4 (1.31%)	2 (0.53%)
CCI ≥ 3	42 (4.7%)	61 (5.5%)	60 (4.9%)
Hospital location and teaching status			
Metropolitan non-teaching	5 (2.23%)	7 (2.2%)	7 (2.1%)
Metropolitan teaching	41 (3.69%)	54 (3.95%)	50 (3.46%)
Non-metropolitan	5 (6%)	9 (8.2%)	9 (7.57%)
Hospital size			
Small	7 (3.1%)	8 (2.8%)	8 (2.35%)
Medium	10 (2.65%)	16 (3.36%)	15 (2.75%)
Large	34 (4.1%)	45 (4.43%)	44 (4.18%)
Insurance			
Medicare	34 (3.18%)	60 (4.39%)	59 (4.05%)
Medicaid	3 (2.4%)	1 (0.78%)	1 (0.69%)
Private insurance	10 (6.11%)	8 (4.01%)	6 (2.9%)
Self-pay	2 (13.84%)	0	0

## Data Availability

This study does not contain copyright material from other sources. Therefore, permission to reproduce copyright materials was not required.
